# Review of Four Refined Clinical Entities in Hereditary Retinal Disorders from Japan

**DOI:** 10.3390/ijms26115166

**Published:** 2025-05-28

**Authors:** Yozo Miyake

**Affiliations:** Nagoya University, 3-707 Suishouenn, Moriyamaku, Nagoya 463-0010, Japan; ymiyake@aichi-med-u.ac.jp; Tel.: +81-052-794-1571

**Keywords:** new clinical entity, hereditary retinal disorder, congenital stationary night blindness (CSNB), complete type CSNB (CSNB1), incomplete type CSNB (CSNB2), total complete bipolar cell dysfunction (CSNB3), occult macular dystrophy, Miyake disease, electrodiagnostic, genetic diagnosis

## Abstract

In the past, only Oguchi disease was reported as a hereditary retinal disease from Japan. Dr. Chuuta Oguch was a Professor of Nagoya University in Japan. During the past 40 years, four new clinical entities in hereditary retinal disorders have been detected by the Miyake group from Nagoya, Japan. All disorders show essentially normal fundi, and the diagnosis was made mainly by the analysis of an electroretinogram (ERG). Gene mutations are detected in three of them. Bipolar cell (BP) dysfunction syndrome: Congenital stationary night blindness (CSNB) with negative ERG (a-wave is larger than b-wave) was named as the Schubert–Bornschein type in 1952 and considered to be an independent clinical entity. In 1986, Miyake group classified ninety patients with the Schubert–Bornschein type into two types (complete and incomplete type). The complete type of CSNB (CSNB1) showed no rod function, but the incomplete type CSNB (CSNB2) showed remaining rod function in both subjective dark adaptation and rod ERG. In order to investigate the pathogenesis, these two types of CSNB were analyzed by comparing the monkey ERGs using different glutamate analogs to the retina. The ERG analysis demonstrated that CSNB1 has a complete functional defect in the ON type BP, while CSNB2 has incomplete functional defects in the ON and OFF type BP in both rod and cone visual pathways. Evidence of several different genetic heterogeneities was reported in both diseases, indicating CSNB1 and CSNB2 are independent clinical entities. Another entity, showing total complete defect of both ON and OFF BP, was detected in 1974 and was reported by Miyake group in a brother and younger sister, showing severe photophobia, nystagmus, extremely low visual acuity, and disappearance of color vision (total color blindness). This disorder is a congenital stational condition, and subjective visual functions were severely deteriorated from birth but remained unchanged through life. This disease was termed “Total complete bipolar cell dysfunction syndrome (CSNB3)”. The relationship between BP and subjective visual function was unknown. These three kinds of BP diseases can provide information on how BP relates to subjective visual functions. Occult macular dystrophy (OMD): Occult macular dystrophy (OMD) was discovered by Miyake group in 1989. This disease shows an unusual, inherited macular dystrophy characterized by progressive decrease visual acuity due to macular dysfunction, but the fundus and fluorescein angiography are essentially normal. The full-field rod and cone ERG do not show any abnormality, but the focal macular ERG (FERG) or multifocal ERG is abnormal and the only method for diagnosis. Many pedigrees of this disorder suggest autosomal dominant heredity, showing a genetic mutation of *RP1L1*. This disease was termed “occult macular dystrophy”. “Occult” means “hidden from sight”. Recently, it has been called “Miyake disease”.

## 1. Background

Reviewing the history of the discovery of hereditary retinal disorders in Japan, Oguchi disease is the only one that was found by Oguchi in 1907 [1]. Oguchi disease is a type of CSNB and an autosomal recessive disease with a genetic mutation of the arrestin gene [2,3] or the rhodopsin kinase gene [4]. The fundus shows a peculiar golden reflex, and the diagnosis is not difficult [5]. Dr. Oguchi was a Professor of Ophthalmology at Nagoya University, Japan.

**Figure 1:**
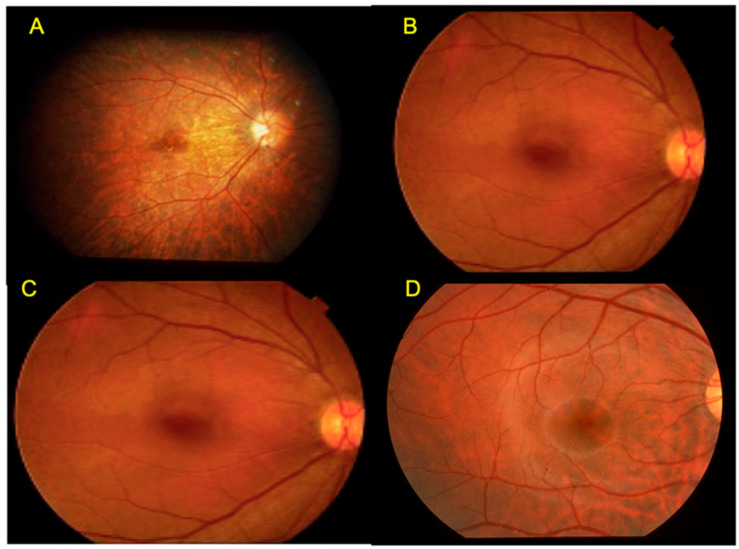
Fundus of complete type congenital stationary night blindness (CSNB1) (**A**) [6]. Incomplete type congenital stationary night blindness (CSNB2) (**B**) [6]. Total complete bipolar cell dysfunction syndrome (CSNB3) (**C**) [10]. Occult macular dystrophy (OMD) (**D**) [11]. All diseases show a normal fundus. (From Miyake [6,10,11]).

In this paper, I summarize four additional newly identified hereditary retinal disorders with normal fundus (Figure 1). We have termed these four diseases as “complete type congenital stationary night blindness (CSNB1)” (A) [6,7,8,9], “incomplete type congenital stationary night blindness (CSNB2)” (B) [6,7,8,9], “total complete bipolar cell dysfunction syndrome (CSNB3)” [10] (C), and “occult macular dystrophy (OMD)” [11,12]. All were reported from Nagoya University.

## 2. Introduction

### 2.1. Congenital Stationary Night Blindness (CSNB) with Normal Fundus: A New Classification

In the past, a type of CSNB with normal fundus was called Schubert–Bornschein type [13], where a unique electroretinogram (ERG) was shown as a negative ERG. In a single bright flash ERG in the dark, the a-wave is normal, but the b-wave is smaller than the a-wave (negative ERG). Since the origin of a-wave is nearly the photoreceptor, while that of the b-wave is the middle retinal layer, negative ERG means middle retinal layer dysfunction, possibly bipolar cell (BP). In 1986, Miyake et al. reported a new classification of CSNB with negative ERG [6]. CSNB with negative ERG (Schubert–Bornschein type) was classified into two types: complete type (CSNB1) and incomplete type (CSNB2), according to the rod function in subjective dark adaptation and rod ERG. CSNB1 lacks rod function completely, but CSNB2 shows some residual rod function [6,7,8,9]. In addition to rod ERG, cone ERG also showed significant differences. Cone ERG looks good in CSNB1, while it looks poor in CSNB2 [6,7,8,9]. Although these two types have common findings, such as a normal fundus, negative ERGs, and normal electrooculograms (EOGs), there are several significant differences, as shown below.

### 2.2. Subjective Dark Adaptation

**Figure 2:**
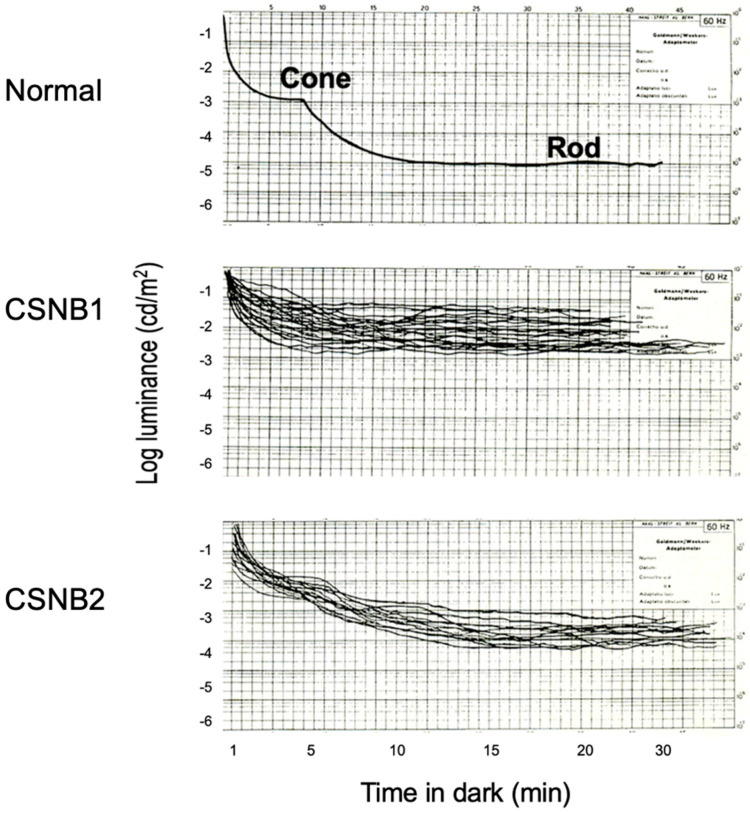
Superimposed subjective dark adaptation curve in a normal patient and CSNB1 and CSNB2 patients. CSNB1 patients lack rod adaptation completely, but CSNB2 patients show preserved rod adaptation [6,8].

Subjective dark adaptation (Figure 2) was tested by a Goldmann–Weekers adaptometer [6,8,9]. The measurements were taken 15° in the superior retina. The superimposed dark adaptation curves in 33 patients with CSNB1 (27 male, 6 female) and 19 patients with CSNB2 (all female) are compared with a normal control.

In CSNB1, the cone threshold is elevated as compared with a normal control, and the rod threshold is absent. In CSNB2, the cone threshold is slightly elevated, and the final threshold of the rod is elevated by approximately 1.0–1.5 log units above normal.

### 2.3. Visual Acuity and Refractive Error

**Figure 3:**
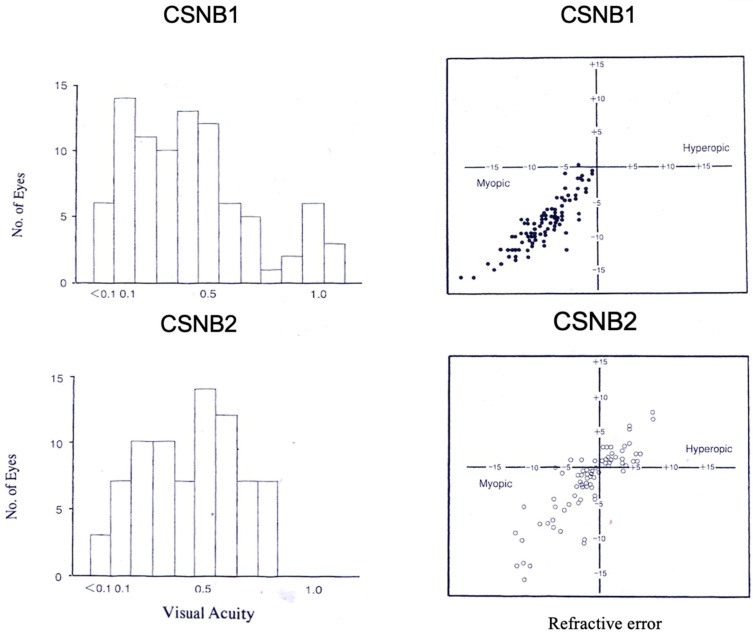
Visual acuity (**left**) and refractive error (**right**) in CSNB1 (**upper**) and CSNB2 (**lower**). Moderately low visual acuity shows in both types of CSNB. Most CSNB1 patients show high to moderate myopia, while CSNB2 patients show both moderate myopia and hyperopia [8]. Visual acuity is shown as decimal visual acuity.

The distribution of the corrected visual acuity of patients with both types is shown in Figure 3. The corrected visual acuity of 88 eyes in CSNB1 (79 male, 9 female) and 80 eyes of CSNB2 (all male) were compared. Both types showed moderately low visual acuity in many patients (CSNB1: mean 0.45, CSNB2: mean 0.49) [8]. These do not differ significantly. However, it should be noted that eight eyes of CSNB1 patients showed normal visual acuity (1.0 or better) [6,8].

The distribution of the refractive errors in the two groups is shown in Figure 3 [6,8]. Most CSNB1 patients have moderate to high myopic refractive error, whereas CSNB2 patients show a wide range of refractive error, from myopia to hyperopia. The mean refractive error was −8.7 diopter in CSNB1 and −2.5 diopter in CSNB2, which is significantly different (*p* < 0.001) [8].

### 2.4. Standard Full-Field ERG (ISCEV Protocol)

**Figure 4:**
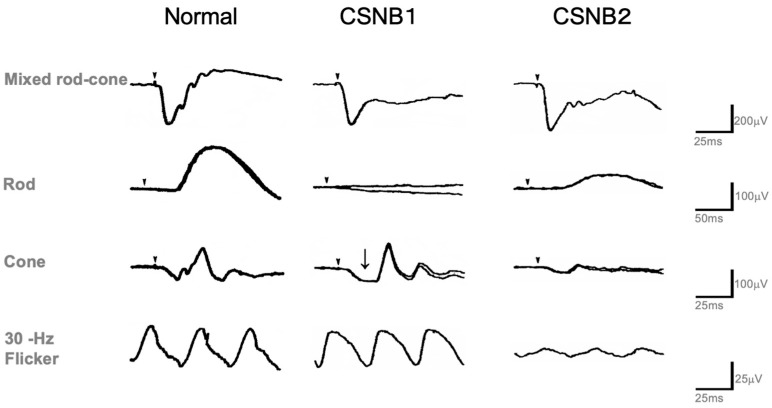
Full-field standard ERGs recorded by the ISCEV protocol in a normal control, CSNB1, and CSNB2. Both types show negative ERG. Rod ERG is absent (CSNB1) but recordable (CSNB2). In spite of the fact that visual acuity shows little difference between the two CSNB, photopic ERGs (cone and 30 Hz flicker) show a significant difference. The cone ERG looks normal in CSNB1, but the bottom of the a-wave shows a plateau shape (arrow). Small arrows in each response indicate stimulus onset.

Figure 4 [9] shows representative examples of the standard full-field ERGs recorded from a normal subject and CSNB1 and CSNB2 patients recorded by the ISCEV protocol [14]. For the diagnosis of both types, standard full-field ERG is the most helpful. Bright flash (mixed rod and cone) ERG recorded after 30 min of dark adaptation shows negative ERG (normal a-wave with extremely reduced b-wave) in both types. Oscillatory potentials are often recordable only in CSNB2. Rod ERG is absent in CSNB1 but recordable (although smaller than normal) in CSNB2. This result is consistent with subjective dark adaptation (Figure 2). On the other hand, cone and 30 Hz flicker ERGs are the function of the cone visual pathway. Both look normal in CSNB1 in terms of amplitude and implicit time. However, the a-wave bottom of the cone ERG is not an acute angle, but it shows a plateau (arrow). This wave shape is caused by the OFF wave, which appears when the ON wave disappears. In CSNB2, both cone and 30 Hz flicker ERG are very deteriorated. This is a key finding of CSNB2 in the differentiation of CSNB1. It also provides a very interesting finding that, in spite of no significant difference in visual acuity between the two types (Figure 3), the ERG of the cone system shows such a big difference. In addition, although the cone threshold in subjective dark adaptation (Figure 2) was elevated more in CSNB1 than CSNB2, cone ERGs look better in CSNB1 than CSNB2.

### 2.5. ON and OFF Responses in Photopic ERG

There are significant potential changes when a light stimulus is turned off. These potentials are called the OFF response, or d-wave, of the ERG. For conventional recordings, a stroboscopic flash is used to elicit the ERGs, and the OFF response is embedded in the ON response. Because there are retinal diseases in which the ON and OFF responses are affected differentially, it became extremely important to record ON and OFF responses separately using a long-duration stimulus [7,15].

**Figure 5:**
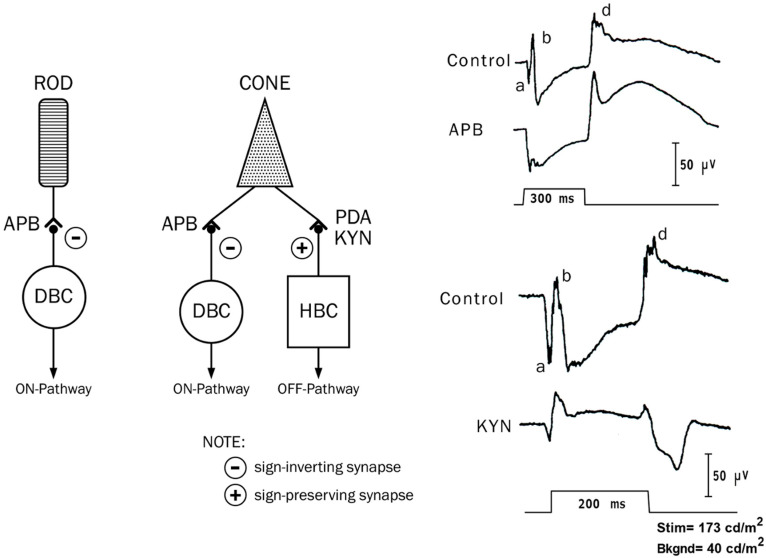
Simplified schema of the rod and cone visual pathway in the mammalian retina [15]. a, b and d on the wave are names of component in ERG.

Figure 5 shows a simplified schema of the rod and cone visual pathways in the mammalian retina (left) and the long-flash photopic ERGs (right), which were shown by Sieving [15]. The photoreceptors transmit visual information to the BP, with the rods containing only depolarizing bipolar cells (DBCs) through sign-inverting (-)synapses (ON synapse). The cones contact both DBCs and hyperpolarizing bipolar cells (HBCs) through sign-inverting (+) synapses (OFF synapse), respectively. The two types of synapses from the photoreceptors to the BPs are selectively sensitive to different glutamate analogs. The sign-inverting synapse (ON synapse) can be blocked by 2-amino-4-phosphobutyric acid (APB) [16]. The sign-preserving synapse (OFF synapse) can be blocked by either cis-2,3-piperidine dicarboxylic acid (PDA) or kynurenic acid (KYN) [17]. Sieving demonstrated the contribution of these glutamate analogs to the DBCs and HBCs to the monkey long-flash photopic ERG [15].

As shown in Figure 5 (right), the control ERG exhibits ON responses (a-waves and b-waves) and OFF responses (d-waves), with a negative plateau between the b-waves and d-waves. By blocking DBC activity, the photopic b-wave was suppressed, and the a- and d-waves were enhanced. After blocking the HBCs with KYN, the a- and d-waves became smaller, and the plateau was elevated above the baseline (Figure 5, arrowheads). Based on the results, it has been proposed that the “push-pull” activity of the HBCs and DBCs is summated in the photopic ERG recorded at the cornea [15]. The a-wave of the photopic ERG evoked by long- and short-duration flashes arises not only from the neural activity of the photoreceptors but also from HBCs. In addition, the b- and d-waves of the photopic ERG elicited by long-duration flashes are produced by an interaction of the HBCs and DBCs, and the cornea-positive peak of the short-flash ERG results from a summation of the b-wave at light onset and the d-wave at light offset [15].

### 2.6. ON and OFF Responses in CSNB

**Figure 6:**
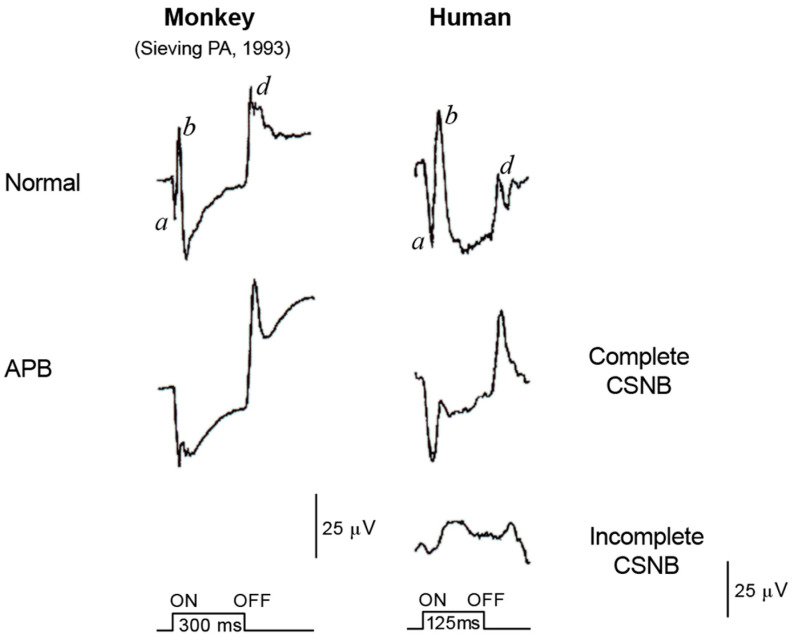
Long-flash photopic ERGs in monkeys are compared with those of patients. Normal responses were compared with APB-treated monkey and CSNB1 and CSNB2 patients. The waveform of CSNB1 is similar to APB-treated monkey ERG [8]. a, b and d on the wave are names of component in ERG.

Using photopic ERGs elicited by long-duration square-wave stimuli, we found that the cone “ON” response generated by depolarizing ON bipolar cells is selectively and severely depressed in patients with CSNB1 [7] (Figure 6). The waveform is similar to that of monkeys after APB is injected into the vitreous to block the ON synapse between photoreceptors and bipolar cells [8].

The OFF response, on the other hand, which is generated by hyperpolarizing bipolar cells, is intact in patients with CSNB1, leading us to hypothesize that the ON function of both the rod and cone visual pathways is completely blocked in eyes with CSNB1. The complete defect of the visual pathway results in complete night blindness, because rods connect only to the ON bipolar cells.

It is interesting to consider why the standard brief flash cone ERG consists of a normal-appearing response (Figure 4) despite the defects of the ON component evaluated by the long-flash photopic ERG in CSNB1. The mechanism involved in generating this phenomenon is as follows.

**Figure 7:**
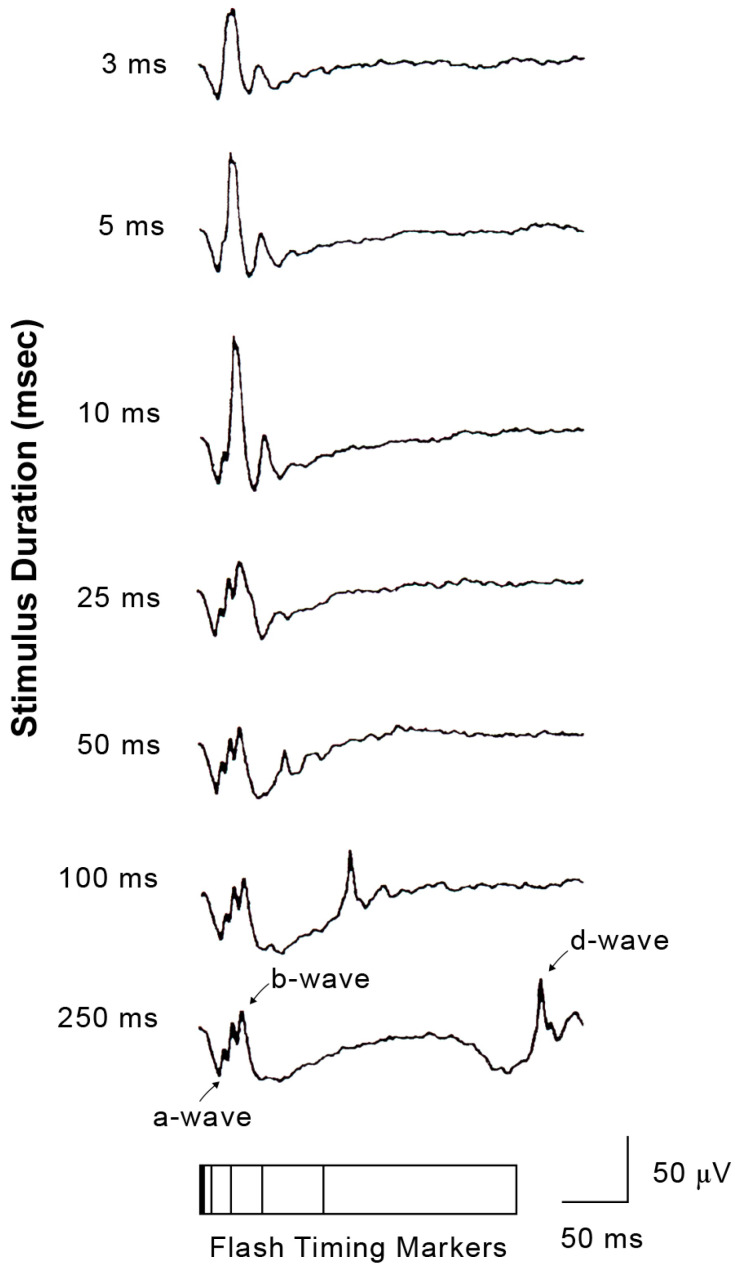
Photopic ERGs recorded with various stimulus durations from a normal subject.

With long-duration stimuli, the a-, b-, and d-waves are clearly separated. As the stimulus duration is short (brief-flash stimuli), the positive component of the photopic ERG consists mainly of d-waves (Figure 7). Therefore, even when the b-wave, a component of the ON response, is absent (as in CSNB1), the d-wave replaces the b-wave, and a positive wave is recorded with brief-flash stimuli.

**Figure 8:**
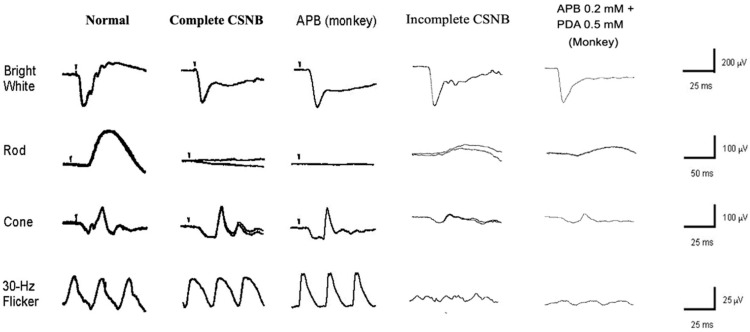
From the left: standard full-field ERGs in a normal human control, CSNB1 patient, monkey model of CSNB1 with APB injected into the vitreous, CSNB2 patient, monkey model of CSNB2 with a small amount of APB and PDA injected into the vitreous. ERGs of CSNB1 and CSNB2 are very similar to each monkey model ERG. Small arrows in each response indicate stimulus onset.

In CSNB2, on the other hand, the story is more complex, with both subnormal ON and OFF responses (Figure 6). We hypothesized that the ON and OFF systems are incompletely disturbed at the level of the bipolar cells in patients with CSNB2. This hypothesis was confirmed by the standard full-field ERGs recorded from the monkey’s eye after being treated with neurotransmitter blocking agents. The technique of full-field ERG recording from monkeys under the same conditions as human patients is shown in Figure 8. The monkey ERGs recorded after the ON synapses were completely blocked by APB were identical to those recorded from CSNB1 patients (Figure 8). After the monkey eye was treated with low levels of APB and PDA to block the ON and OFF synapses incompletely, the shape of the full-field ERG is similar to that for CSNB2 (Figure 8). These results indicate that a regular full-field ERG recording (ISCEV protocol [14]) can provide the ON and OFF BP functions.

### 2.7. Short-Wavelength Cone (S-Cone) ERG and Subjective Blue Sensitivity

**Figure 9:**
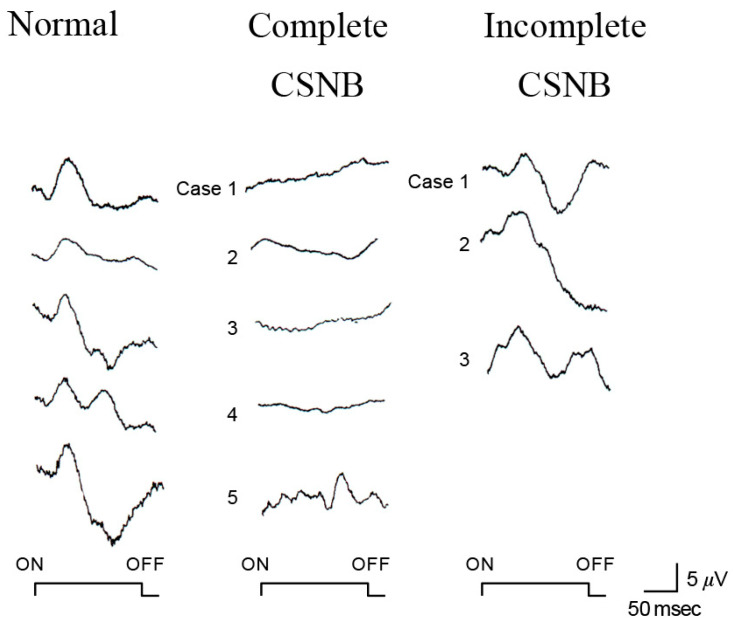
Full-field S-cone ERGs in normal subjects and CSNB1 and CSNB2 patients. S-cone ERG is absent in CSNB1 but subnormal in CSNB2 patients.

The S-cone ERGs are markedly different in the two types of CSNB. Because the S-cones connect only to the ON BC, whereas middle- and long-wavelength cones (LM-cones) connect to both ON and OFF BCs [18]. Thus, the S-cone function should be absent in CSNB1. Indeed, the full-field S-cone ERG was nonrecordable in CSNB1 patients (Figure 9).

**Figure 10:**
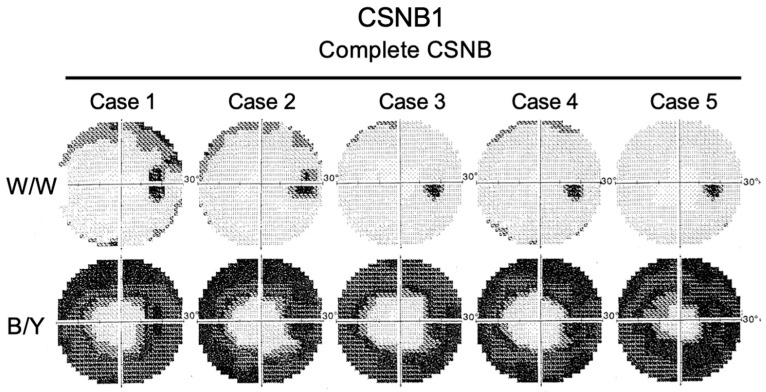
Two-color perimetry in white-on-white (W/W) and blue-on-yellow (B/Y) in 5 patients with CSNB1. In B/Y perimetry, only the central zone is preserved [21].

However, surprisingly enough, the subjective blue sensitivity is normal in CSNB1 [19,20]. Two-color perimetry was performed to evaluate the subjective S-cone function of the entire retina in five patients with CSNB1 (Figure 10) [21].

White-on-white (W/W) perimetry was essentially normal, but blue-on-yellow (B/Y) perimetry showed diffuse dysfunction of S-cones; however, only the central field was preserved. The blue sensitivity is nearly normal only in the central 10° to 15° in CSNB1 [21]. Psychophysically determined color vision is influenced mainly by the central visual field. These findings lead us to believe that the macula of CSNB1 patients must have a unique pathology that is different from that in other parts of the retina in CSNB1.

**Figure 11:**
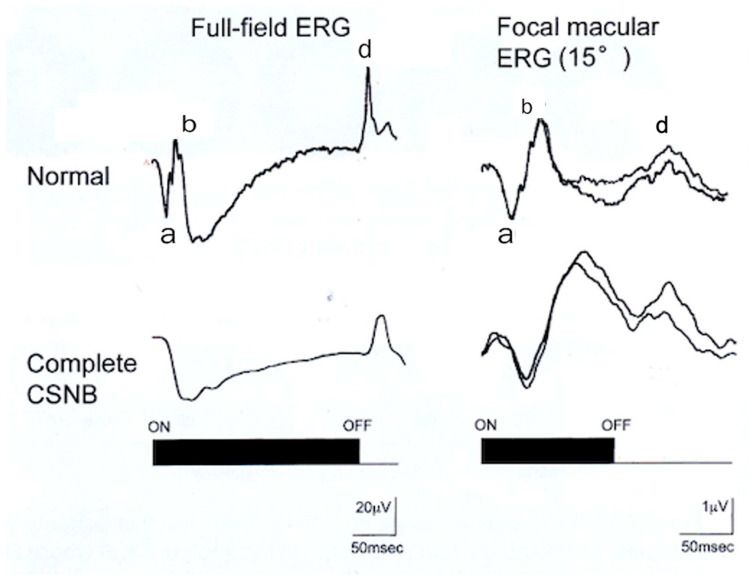
Long-flash photopic full-field ERG (**left**) and focal macular ERG (**right**) in a normal subject (**upper**) and a patient with CSNB1 (**lower**) [9]. a, b, and d on the wave are components of ERG.

To solve this interesting finding, the focal macular ERG (FERG) was recorded with a 15° spot from the macula of CSNB1. Full-field photopic ERG and FERG recorded by long-flash stimuli in a CSNB1 patient (Figure 11) [9]. ON and OFF responses were separately recorded.

In a normal control, a-wave, b-wave, oscillatory potentials (OPs), and OFF wave (d-wave) are shown in Figure 11. In CSNB1, the full-field ERG shows that the a-wave and d-wave are clearly recordable, but the b-wave is extremely small, and OPs are absent. In focal macular ERG, in addition to an a-wave, a big b-wave is also recorded in CSNB1. The wave shape of focal macular ERG is quite different from that of full-field ERG. The amplitude of the b-wave is normal; however, the implicit time of the b-wave is far delayed.

The implicit time of the b-wave is similar to that of the S-cone ERG in normal subjects. This result may indicate that only the macular function of the S-cone may still be alive in CSNB1.

### 2.8. Molecular Genetics

Since we reported a new classification of CSNB in 1986 [6], genetic research has been performed by several institutes to prove that CSNB1 and CSNB2 are different clinical entities [22]. Evidence of genetic heterogeneity in X-linked (XR) and autosomal recessive (AR) CSNB has been reported by several authors. In XR CSNB2, *CACNA1F* was reported [23,24,25,26]. In XR CSNB1, *NYX* was reported [27,28]. In AR CSNB1, *GRM6* [29], *TRPM1* [30,31,32], *GPR179* [33], *GRM6* [29], and *LRIT3* [26,34] were reported. In AR CSNB2, *CABP4* [35,36,37,38,39,40,41,42] and *CACNA_2_D_4_* [43] were reported. Regardless of the mutated gene, CSNB1 always shows BPs are affected by involvement in signal transmission from rods or the unsuitable localization of ion channels in BPs. On the other hand, CSNB2 always shows calcium channel problems. This gene searching information was obtained from phenotype analysis, mainly by ERG.

### 2.9. Total Complete Bipolar Cell Dysfunction Syndrome (TCBDS)

We detected two clinical diseases of BPs, CSNB1 and CSNB2, which showed different BC dysfunctions. By analyzing the pathophysiology of these diseases, we could determine the correlation between psychophysical and electrophysiological abnormalities in relation to BPs.

In 1980, Miyake et al. reported a brother and a sister who showed complete dysfunction of both ON and OFF BCs severely from birth [10]. We followed these patients for longer than 34 years and found that the condition is stationary. We termed this disease as total complete bipolar cell dysfunction syndrome (TCBDS) (CSNB3).

**Figure 12:**
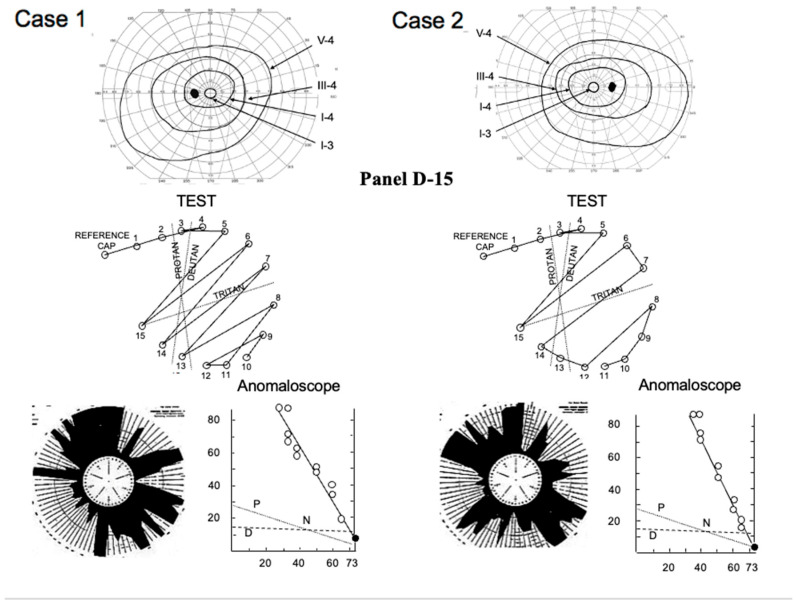
Visual fields and color vision in two patients with TCBDS (CSNB3). Panel D-15 test, 100 Hue Test and Nagel Anomaloscope show similar results of total color blindness (rod monochromat). P: protan, D: deutan, N: normal (from Miyake et al. [10]).

A pair of siblings (5-year-old girl and 7-year-old boy) visited our clinic in 1971. Both suffered severe photophobia, extremely low visual acuity (0.04~0.06), nystagmus, and total color blindness (Figure 12). The fundus and fluoresceine angiography were essentially normal (Figure 1). Neither complained of night blindness. The first impression was rod monochromat because of the color vision tests (Figure 12). The results of the Panel D-15 Dichotomous Color Vision Test demonstrated a characteristic scotoma pattern. The 100 Hue Test developed by the Japan Color Research Institute (JCR) did not reveal any characteristic axis, but it indicated a severely impaired color discrimination ability. The Nagel Anomaloscope results indicated a characteristic matching range consisted with rod monochromacy. However, the full-field ERG showed a negative shape with nonrecordable rod ERG and cone ERG. Taking the bipolar cell functions of CSNB1 and CSNB2 into consideration, the results from these two patients strongly suggest complete dysfunction of ON and OFF BPs. As far as I know, the mutated gene has not been reported in the past. Since many CSNB2 patients who show calcium channel abnormalities reveal ON and OFF BP problems, this disease can be one of them [42]. However, no patient has been reported to have shown absent rod and cone ERG in CSNB2.

**Figure 13:**
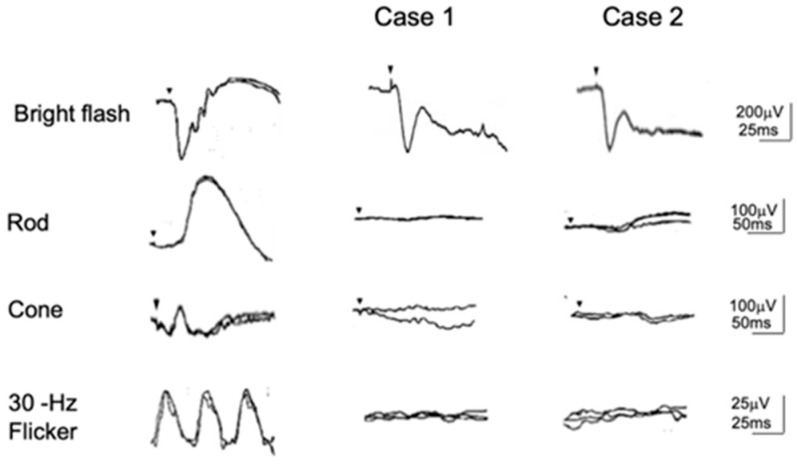
Full-field ERG in 2 patients with TCBDS (CSNB3). Bright-flash ERG shows a negative shape. Both rod and cone ERGs are completely absent [10]. Arrow indicates stimulus onset.

**Figure 14:**
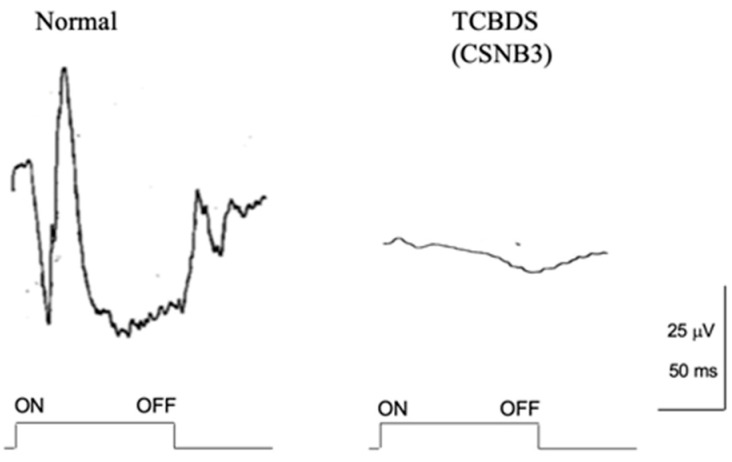
Photopic long-flash full-field ERG in a normal subject (**left**) and TCBDS (CSNB3) (**right**) [10].

These two patients were followed for 34 years, and several examinations were performed during this time. In 1983, the ISCEV protocol was used for ERG testing (Figure 13). The absent rod ERG with negative ERG is similar to CSNB1; however, cone and flicker ERGs were completely absent. The photopic long-flash ERG was nearly nonrecordable (Figure 14), indicating severe dysfunction of ON and OFF BCs.

These results strongly suggest that ON and OFF BCs are both completely impaired in both the rod and cone visual pathways. In the case where the BC function is gone, it is reasonable that almost the entire visual function is wiped out. Indeed, the visual function, including very poor visual acuity, total color blindness, severe photophobia, and nystagmus, looks like rod monochromat (Figure 12). However, one mystery was that neither patient complained of night blindness despite a nonrecordable rod ERG (Figure 13). Night blindness is congenital, and the photophobia is such that the patient may not become severely aware of night blindness. Another surprise was that the Goldmann–Weeker visual field did not show severe abnormality (Figure 12).

**Figure 15:**
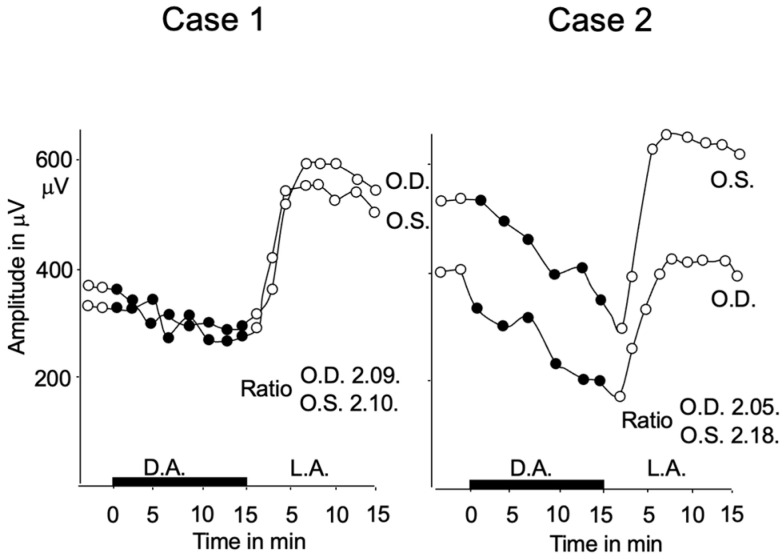
EOG in two patients with CSNB3. EOG is normal.

The EOG is completely normal (Figure 15). It is interesting that the EOG in all three DC diseases mentioned above is normal. This result suggests that the BC function does not significantly influence the retinal pigment epithelium function.

The above three diseases are all hereditary, stationary disorders with BC dysfunction. One can assume what kind of BC is disturbed and how the visual function may change accordingly.

### 2.10. Occult Macular Dystrophy

In addition to the three kinds of hereditary retinal diseases of BC dysfunction mentioned above, where the fundus is normal, one more hereditary macular disease with normal fundus appearance was included. From 1976, Miyake started to build a focal macular ERG (FERG) with Dr. Tatsuo Hirose in Boston [44]. After long-term efforts to build the FERG, the Miyake group succeeded in making the most informative system under the fundus monitor through an infrared fundus camera (Cannon) in 1988 [45,46]. By using this system, most ERG components could be precisely recorded. They included a-wave, b-wave, oscillatory potentials, OFF d-wave, and PhNR [45,46].

**Figure 16:**
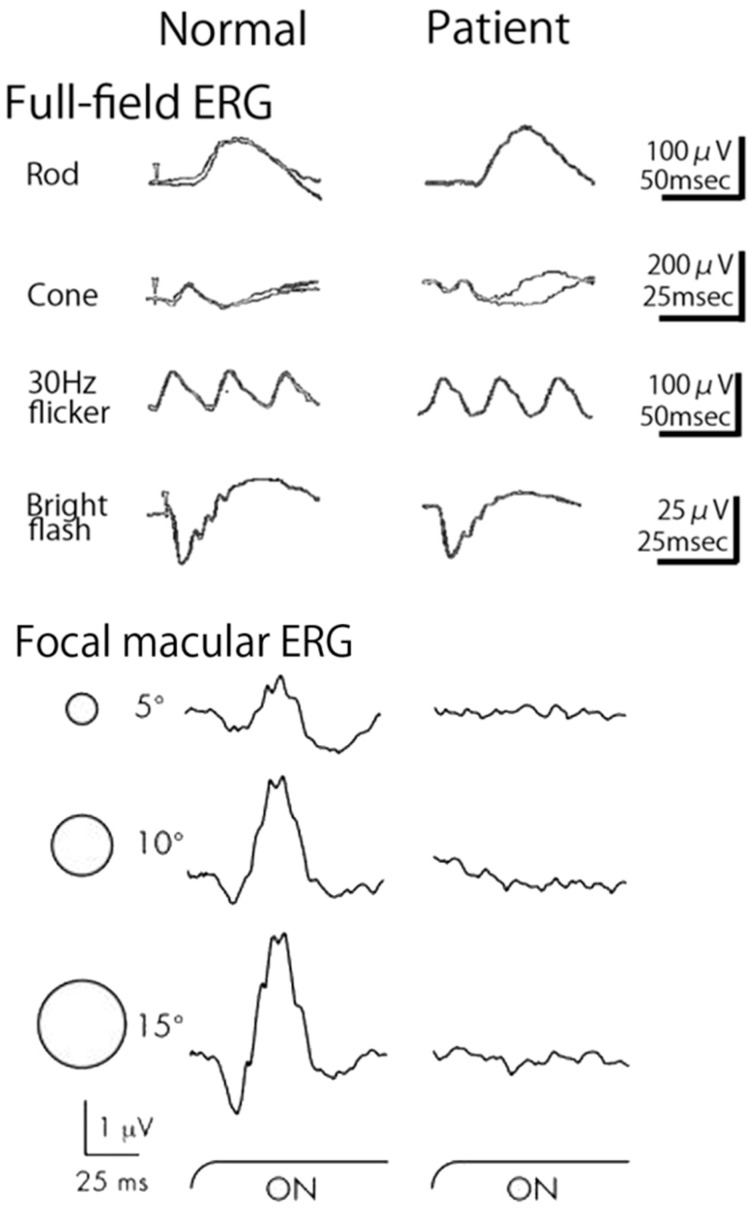
Full-field ERGs (**left**) and focal macular ERG (FERG) recorded by 3 different sizes of stimulus spots (5°, 10°, 15°) in diameter (**right**) in a normal subject and a patient with occult macular dystrophy (OMD). Full-field ERG is normal, but FERG is very abnormal. Arrow indicates stimulus onset.

The Miyake group tried to record FERG from many patients and found a new disease, “occult macular dystrophy (OMD)” [11,12]. Our first patient (28-year-old-woman) complained of a gradual decrease in visual acuity (0.3~0.4) during the past 10 years in both eyes. The fundus and fluorescein angiography were normal. The full-field ERGs were normal, but the FERG was enormously abnormal, indicating that only the macula is abnormal. Other family members were examined, and another two members of this family (24-year-old-brother, 54-year-old-father) showed similar abnormalities. The ERG findings in a representative patient with OMD are shown in Figure 16.

**Figure 17:**
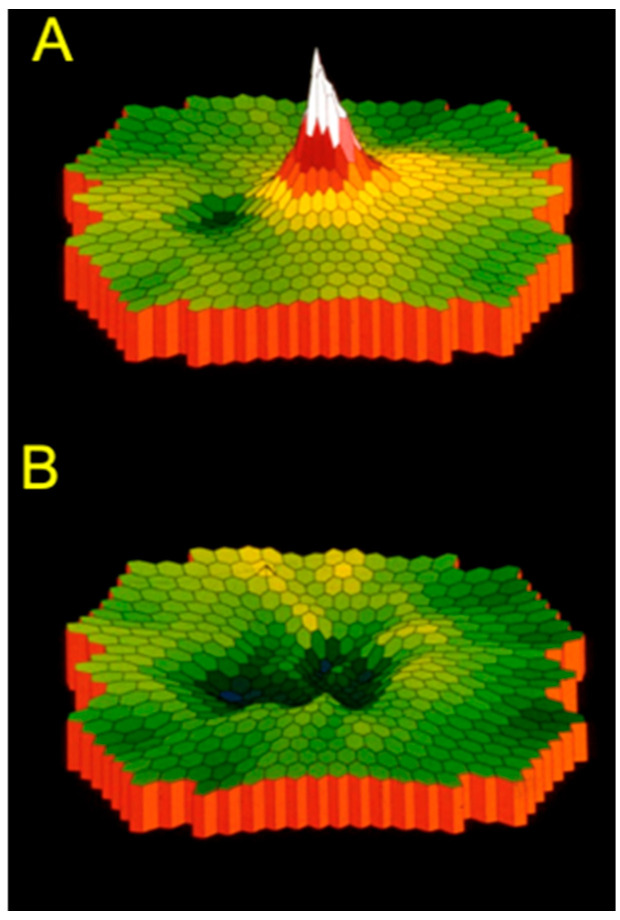
Multifocal ERG in a normal subject (**A**) and occult macular dystrophy (OMD) (**B**). Only the central retina is depressed, and the other part is normal.

The full-field ERGs (left) and the FERGs (right) of three different spot sizes (5°,10°, 15° in diameter) are shown in a representative OMD patient in Figure 16. The full-field ERG is normal, but the focal macular ERGs are very abnormal. The multifocal ERG [47] also shows clearly the pathology of the affected retina (Figure 17).

**Figure 18:**
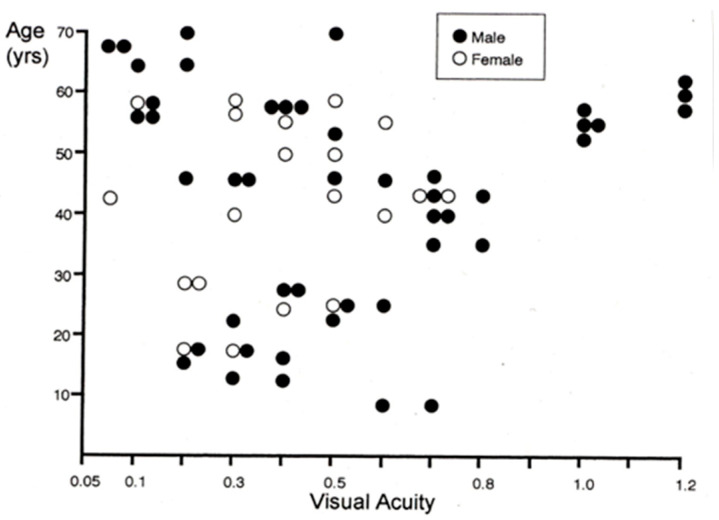
Corrected visual acuity of OMD and age of the patients. There is little correlation between visual acuity and age. Visual acuity is shown as decimal visual acuity.

In 1996, Miyake et al. reported 16 patients with this disease and analyzed them from several points of view [12]. The disease name was termed as “Occult macular dystrophy (OMD)” [12]. “Occult” means “hidden from sight”. The distribution of visual acuity and the age of OMD patients are shown in Figure 18. Visual acuity shows a large variation and little correlation with age [48].

**Figure 19:**
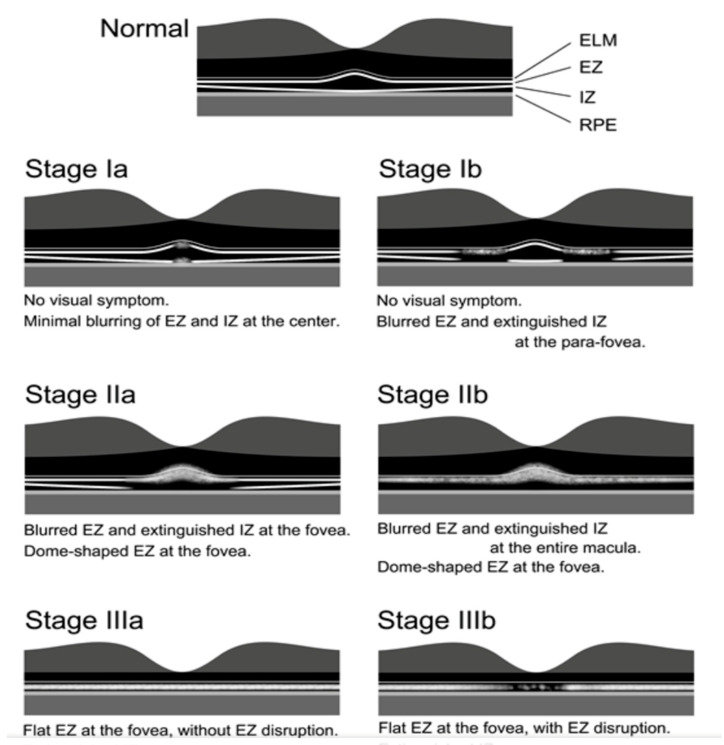
OCT and progress of OMD [51]. ELM: external limiting membrane, EZ: ellipsoid zone, IZ: internal segment ellipsoid, RPE: retinal pigment epithelium.

OCT provides some information as to the progress of OMD [49,50,51] (Figure 19). The photoreceptor structures at the fovea and parafovea region determined by OCT gradually changed as the duration of OMD increased. Nakamura et al. graded the OCT changes in the photoreceptor layers in 61 cases. All of the patients at stage 1 were asymptomatic with good visual acuity. Stage 11 was the largest group among OMD patients; disease duration ranged from 1 to 53 years. Stage III was the late stage of OMD; the disease duration ranged from 6 to 65 years, and the corrected visual acuity of stage III b ranged from 0.08 to 0.31.

**Figure 20:**
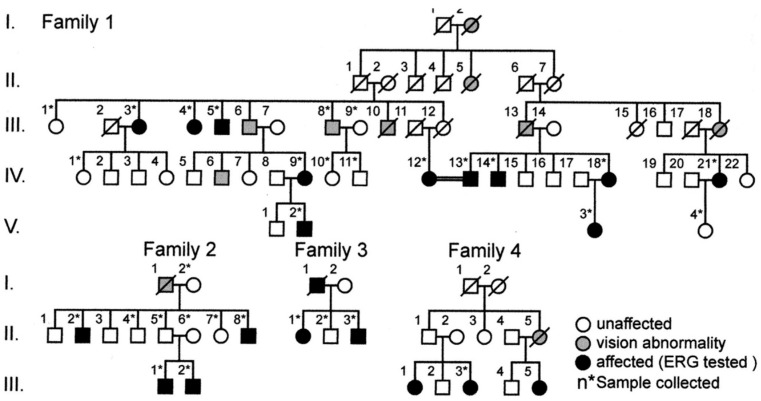
Examples of OMD families with *RP1L1* gene mutation. From analysis of Family 1, *RP1L1* gene mutation was found for the first time [47,48].

Thereafter, a lot of attention was paid to detecting gene mutations. The hereditary mode of this family is autosomal dominant, and in 2010, a mutation of the *RP1L1* gene after gene analysis of a large family [52] was found (Figure 20, Family 1) by the Miyake group.

The *RP1L1* gene was originally identified by human and mouse genome sequencing, and it contained four exons that span 50 kb on chromosome 8p. Immunohistochemistry showed that it is expressed in the rod and cone photoreceptors of cynomolgus monkeys [53]. The *RP1L1* protein was suggested to be involved in the morphologic and functional maintenance of photoreceptors [54,55]. The cellular mechanisms that explain why only the macular region is impaired in human OMD patients have not been determined.

In the Japanese population, *RP1L1* gene mutations are rarely found in sporadic cases [56,57]. There are OMD families without the *RP1L1* mutation where autosomal recessive inheritance is assumed. The genetic background leading to the OMD may be a variant, and other genetic causes will probably be determined in the future.

The Miyake group developed the focal macular ERG system in 1986 after long-term efforts. This focal macular ERG system is very informative [45,46], and this system was in the forefront of the multifocal ERG developed by Sutter in 1994 [47]. By using this method, OMD was discovered in 1989 [11]. In 2010, the gene mutation of OMD, *RP1L1*, was detected by Akahori et al. [52].

Accordingly, the development of diagnostic instruments, the discovery of new diseases, and the discovery of gene mutations are all achievements completed by Miyake’s group, which may be only one brilliant feat in medical history. Because of the substantial contribution of our group, OMD diseases with the RP1L1 gene mutations are now referred to as “Miyake disease” [57,58,59]. In 2015, the Japanese Ministry of Health, Labor and Welfare for medical care declared Miyake disease as one of the limited diseases for priority functional support.
